# Influence of previous experience with and beliefs regarding anal cancer screening on willingness to be screened among men living with HIV

**DOI:** 10.1186/s12889-022-14471-4

**Published:** 2022-12-28

**Authors:** Jennifer L. Gillis, Troy Grennan, Ramandip Grewal, Gina Ogilvie, Mark Gaspar, Daniel Grace, Aisha Lofters, Janet M. Raboud, Olli Saarela, Paul MacPherson, Ron Rosenes, Irving E. Salit, Ann N. Burchell, for the OHTN Cohort Study Team, Abigail Kroch, Ann Burchell, Sergio Rueda, Gordon Arbess, Jeffrey Cohen, Curtis Cooper, Elizabeth Lavoie, Fred Crouzat, Nisha Andany, Sharon Walmsley, Michael Silverman, Roger Sandre, Wangari Tharao, Holly Gauvin, Fiona Smaill

**Affiliations:** 1 Dalla Lana School of Public Health, University of Toronto, Toronto, Ontario Canada; 2 Toronto General Hospital Research Institute, University Health Network, Toronto, Ontario Canada; 3 MAP Centre for Urban Health Solutions, Li Ka Shing Knowledge Institute, St. Michael’s Hospital, 209 Victoria St, Toronto, ON M5B 1T8 Canada; 4 British Columbia Centre for Disease Control, Vancouver, British Columbia Canada; 5 Faculty of Medicine, University of British Columbia, Vancouver, British Columbia Canada; 6 Women’s College Research Institute, Women’s College Hospital, Toronto, Ontario Canada; 7 The Ottawa Hospital Research Institute, The Ottawa Hospital, Ottawa, Ontario Canada; 8 Department of Epidemiology and Public Health, Faculty of Medicine, University of Ottawa, Ottawa, Ontario Canada; 9 Department of Family and Community Medicine, Faculty of Medicine, University of Toronto, Toronto, Ontario Canada

**Keywords:** HIV, Men living with HIV, Anal cancer, Cancer screening, Anoscopy, Human papillomavirus (HPV), Patient acceptance of health care

## Abstract

**Background:**

Implementation of anal cancer screening requires the procedure to be acceptable to the target population. Our objective was to assess the beliefs of men living with HIV regarding anal cancer screening and identify factors associated with their willingness to participate in screening.

**Methods:**

We developed a cross-sectional questionnaire using the Theory of Planned Behavior to examine beliefs regarding prevention of human papillomavirus (HPV)-related diseases, administered to men living with HIV in 2016–2017 in a multi-site HIV clinical cohort. Correspondence analysis was used to examine the interrelationships between men’s beliefs and willingness to undergo anal cancer screening. We used multivariable proportional odds models to identify factors associated with increasing willingness. Results were reported as adjusted odds ratios (aOR) with 95% confidence intervals (CI).

**Results:**

Among 1677 male participants, the vast majority (90%) would be willing to undergo screening by “anal Pap test”; willingness clustered with positive beliefs (e.g. confident they can get screened; disagree that they will feel pain) in the correspondence analysis. Higher self-perceived risk for anal cancer and positive beliefs regarding screening were associated with higher willingness to be screened. Gay, bisexual and other men who have sex with men had higher willingness (aOR = 1.62; 95% CI: 1.15, 2.29) than heterosexual men. Racialized men reported lower willingness (aOR = 0.68; 95% CI: 0.54, 0.89) than white men.

**Conclusions:**

Men generally had positive beliefs and were willing to undergo screening, though there were differences by sexual orientation and racial identity. Tailored community-led initiatives could focus on men’s understanding of their risk and expectations of anal cancer screening to facilitate participation.

**Supplementary Information:**

The online version contains supplementary material available at 10.1186/s12889-022-14471-4.

## Background

Men living with HIV have a high burden of human papillomavirus (HPV)-associated disease, particularly anal cancer. In a recent systematic review, incidence rates for anal cancer in this population were 32 per 100,000 for HIV-positive men who have sex with women and 85 per 100,000 for HIV-positive men who have sex with men [1]. These rates are magnitudes higher than those of the general male population, where anal cancer incidence is less than 2 per 100,000 [1]. These rates are also notably higher than the incidence of cervical cancer prior to the adoption of cervical cancer screening programs [1, 2], which have led to significant reductions in the incidence rates and mortality of cervical cancer [2]. Due in part to the success of such programs, anal cancer prevention efforts have explored the potential for screening modalities that are similar to those used in cervical cancer screening, namely anal swabs for cytology (“anal Pap tests”) and HPV testing for initial screening and high resolution anoscopy (analogous to colposcopy) for diagnosis of anal pre-cancer; however, clinical guidelines are needed [3, 4]. The success of cervical cancer screening is not only related to the clinical utility of the screening tests but is directly linked to the extent of coverage and participation in the programs [2]. It is imperative that possible barriers and facilitators to anal cancer screening are identified to ensure implementation of any developed guidelines encourages high levels of participation and equitable access to care.

Some studies have quantified uptake and participation in anal cancer screening, where uptake was highest when screening was offered for free as part of research studies conducted in multi-payer healthcare settings [5–10]. This research has predominantly focused on identifying ‘who’ is accepting and being screened for anal cancer, with most studies limited to study populations of gay, bisexual and other men who have sex with men [5–9]. To date, few studies have examined real-world uptake more broadly for men living with HIV in general clinical practice. In a recent study in single-payer healthcare setting, notable disparities in self-reported receipt of anal cancer screening were observed, where men living with HIV from some racialized groups were less likely to have discussed anal cancer screening with a healthcare provider or to have had anal Pap or anoscopy [10]; moreover, heterosexual men were less likely to have discussed screening or to have been screened [10]. Further exploration is needed elucidate factors contributing to these observed differences. Specifically, few studies have evaluated factors that influence the beliefs and willingness of men living with HIV to participate in anal cancer screening [9, 11]. Although studies have shown the screening modalities to be tolerable (e.g. minimal psychological distress, pain) [5, 12–14], no studies have evaluated how prior experience with screening may modify the role of men’s beliefs in influencing their willingness to be screened in the future.

### Aim

To support patient-provider clinical decision-making and participation in screening, we aimed to identify factors that influence acceptability of anal cancer screening among men living with HIV. Using data from an ethno-racially diverse cohort of men living with HIV, our objective was to assess these men’s beliefs regarding anal cancer screening and identify factors associated with willingness to participate in screening in a single-payer healthcare setting. We hypothesized that previous experience with anal cancer screening would influence men’s beliefs regarding screening and alter the association of these beliefs with willingness to be screened.

## Methods

### Data source and study design

This was a cross-sectional analysis of data from participants of the Ontario HIV Treatment Network (OHTN) Cohort Study (OCS) collected at their annual interview between April 2016 and June 2017. The OCS is a voluntary, HIV clinical cohort of people living with HIV who are at least 16 years of age with the ability to give informed consent and who receive care at one of nine HIV clinics in Ontario, Canada [15]. Participants who were receiving clinical care at the OCS sites were considered under active follow-up and completed annual interviewer-led questionnaires, which collected socio-demographic, behavioural and psychosocial measures [15]. Clinical data on CD4 count, viral load and comorbidities are abstracted from medical charts [15]. For our analyses, sexual orientation was categorized as gay-identified, bisexual-identified, non-gay or bisexual-identified men who have sex with men (forthwith referred to as ‘other men who have sex with men’), and heterosexual-identified men who have not reported sex with other men. Race was categorized as Indigenous; African, Caribbean, Black; Asian; white; Latin American; multiple races. Men were considered to be from racialized groups in Canada, as per the Ontario Human Rights Commission [16], if they identified as Indigenous; African, Caribbean or Black; Asian; Latin American; or multiple races.

### HPV Questionnaire Module & key Measures of beliefs and willingness

A questionnaire module examining men’s knowledge, experience and beliefs regarding HPV and associated disease, vaccination and screening was active and administered between April 2016 and June 2017 as part of the annual OCS questionnaire. During that period, the HPV questionnaire module was administered to all interviewed OCS participants who self-identified as men (cis- and transgender). We developed the module based on previous literature and published questionnaires [8, 17–25], examining HPV and associated disease prevention. We applied the Theory of Planned Behavior [21, 26, 27] to design the statements that assessed men’s beliefs regarding anal cancer screening. This theory postulates that individuals are more likely to participate in screening when they have positive beliefs regarding the process [27]. We asked men to assess their beliefs regarding anal cancer screening through seven statements related to the three theoretical constructs: 1) normative expectations (2 statements: “*My doctor thinks that I should get an exam for anal cancer*”, “*In general, people who are important to me would encourage me to get an exam for anal cancer*”); 2) perceived self-efficacy (2 statements: “*I can find out where to go to get an exam for anal cancer*”, “*I am confident that I could get an exam for anal cancer in the next year, if I chose to*”); and 3) behavioral beliefs regarding likely outcomes (3 statements: “*I will feel pain during the procedure*”, “*I have a high chance of getting unpleasant short-term side effects, like pain or bleeding, after the procedure*”, “*If anal pre-cancer is found, I will be offered treatment*”). Men could respond to each statement using a 5-point Likert response format of “strongly agree”, “agree”, “neither agree nor disagree”, “disagree”, and “strongly disagree”, or respond “don’t know” or refuse to answer. Men who agreed with these statements were considered to express positive beliefs, with the exception of the statements on pain and side effects where disagreement was considered positive.

We measured men’s willingness to be screened by asking “*Think about what you might do in the next year. If anal cancer screening were offered to you, would you get an exam where a doctor or nurse inserts a swab (like a long, thin Q-tip) into your anus (“anal Pap test”)*.” Using a 5-point Likert response format, men could respond “very likely”, “likely”, “undecided”, “unlikely”, “very unlikely”, and “don’t know” or refuse to answer; men who responded with “don’t know” were reclassified as “undecided” for analysis. Pertinent questions from the HPV module available in Supplemental Table 1.

### Statistical methods

Our main outcome of interest was men’s willingness to undergo an “anal Pap test”. We used correspondence analysis [28] to assess the interrelationships between the components of men’s beliefs regarding anal cancer screening and the association of these beliefs with willingness to be screened. This method is an exploratory, multivariate graphical technique that examines the relationships between levels of categorical variables [28]. Supplementary variables, such as sociodemographic factors, were projected onto the graphical output of the correspondence analysis to assess their relationship to men’s beliefs and willingness [28]. Results informed the operationalization of measures for the proportional odds model analysis, where beliefs were categorized as “strongly positive”, “positive”, and “negative/neutral”, and willingness was categorized as “very likely”, “likely”, and “unlikely/undecided”.

Using the Theory of Planned Behavior to guide model development, the multivariable proportional odds model [29] examined the association of beliefs and sociodemographic factors with willingness to undergo anal cancer screening by “anal Pap test”, categorized as “very likely”, “likely”, and “unlikely/undecided”. Additionally, pre-disposing (e.g., age, sexual orientation, race, and history of screening), enabling (e.g., HPV awareness, comfort discussing anal health), and need-based (e.g., previous diagnosis with anogenital warts) factors of the Andersen Behavioral Model were included in the model, as was perceived susceptibility, which is a construct of the Health Belief Model and need-based factor of the Andersen Model. Results are presented as adjusted odds ratios (aOR) and 95% confidence intervals (95% CI). We assessed the proportional odds assumption graphically for each factor and using the score test for the multivariable model. We tested our hypothesis that past experience with screening modifies the relationship between men’s beliefs regarding screening and willingness using a single global likelihood ratio test; the joint importance of all interactions between past experience with screening and beliefs were tested, where all interactions were retained if significant [30]. All analyses were conducted using SAS 9.4 (SAS Institute, Inc., Cary, North Carolina, USA).

### Research ethics approval

Research ethics approvals for the questionnaire module and this study were received from the institutional review boards of participating centers.

## Results

A total of 1677 men completed the HPV questionnaire module between April 2016 and June 2017. Over 99% of men who completed the questionnaire self-identified as cisgender. Seventy-two percent (72%) were gay, 7% bisexual, 5% were other men who have sex with men, and 16% heterosexual (Table 1). The median age (interquartile range, IQR) was 53 (45–59) and 70% of men identified as white, 11% African, Caribbean or Black, 7% Asian, 5% Latin American, 4% Indigenous and 4% as multiracial. In total, 40% of men reported previous anal cancer screening by anal cytology or anoscopy; in comparison to the overall sample, the majority of men previously screened were gay, white, and living with HIV longer (Table 1).

**Table 1:** Characteristics of men living with HIV who completed the human papillomavirus (HPV) questionnaire module in 2016–2017 in the Ontario HIV Treatment Network Cohort Study (OCS), overall and among those who have had anal Cytology or anoscopy (i.e., previously screened)

***Demographic Factors***	Overall Sample (***n =*** 1677)		Has had anal Pap or anoscopy (Previously Screened) (***n =*** 659)	
**Age**, Median (P25-P75)	53	(45–59)	54	(48–60)
**Sexual Orientation**				
Heterosexual / Straight	261	16%	29	4%
Gay	1208	72%	562	84%
Bisexual	116	7%	37	6%
Other men who have sex with men	82	5%	27	4%
**Race**				
White	1164	70%	513	76%
African/Caribbean/Black	183	11%	43	6%
Asian	115	7%	24	4%
Latin American	79	5%	28	4%
Indigenous	62	4%	22	3%
Multiracial	65	4%	23	3%
**Education**				
Less than high school	181	11%	48	7%
Completed high school	281	17%	103	15%
Some post-secondary	285	17%	119	18%
Completed post-secondary	929	55%	389	58%
**Gross Personal Income**				
Less than $20,000	599	36%	208	31%
$20,000 to $39,999	377	23%	166	25%
$40,000 to $59,999	260	16%	93	14%
$60,000 to $79,999	188	11%	84	13%
$80,000 to $99,999	93	6%	36	5%
$100,000 or more	128	8%	62	9%
**Year Diagnosed with HIV**, Median (P25-P75)	2001	(1992–2009)	1998	(1991–2007)
**Years with HIV (diagnosed)** Median (P25-P75)	15	(7–23)	19	(9–25)
**HIV Risk Factor (Hierarchical)**				
MSM	1260	75%	565	84%
MSM-IDU	111	7%	50	7%
IDU	79	5%	13	2%
From country with generalized HIV epidemic	84	5%	7	1%
Heterosexual (partner with identified risk)	85	5%	10	1%
Other/No identified risk	58	5%	14	2%
**Ever on Antiretroviral Therapy**	1649	98%	649	97%
**Self-Reported Previous Diagnosis of AIDS**	311	19%	151	23%
**Familiar with HPV**	851	52%	440	67%
**Knows someone with HPV-associated cancer**	200	12%	117	18%
**Comfortable discussing anal health with doctor**	1360	84%	585	89%
**Perceived lifetime chance of anal cancer**				
Don’t know	205	13%	74	11%
No chance	317	20%	90	14%
Low chance	717	44%	265	40%
Moderate chance	269	16%	142	22%
High	68	4%	45	7%
Certain I will get it or have it	50	3%	43	7%

*HPV* Human papillomavirus, *MSM* Men who have sex with men, *IDU* Injection drug use

Generally, men had positive beliefs regarding anal cancer screening (Table 2). Regarding **normative expectations**, most men believed that people important to them would encourage them to be screened (13% strongly agreeing and 62% agreeing); however, men were less certain about their doctor’s recommendation; only 31% believed that “[*their] doctor thinks that [they] should get an exam for anal cancer*”. The vast majority of men had positive beliefs regarding their **self-efficacy**: 90% believed that they could find out where to get screened and 88% were confident they could get screened in the next year if they chose to. Similarly, over 90% of men were confident they would be offered treatment if anal pre-cancer were found. Although some men were concerned about pain and side effects related to screening, about half believed that they would *not* experience pain (51%) or side effects (48%).

**Table 2:** Men’s beliefs regarding anal cancer screening and willingness to undergo screening among men living with HIV attending HIV specialty care in 2016–2017 in the Ontario HIV Treatment Network Cohort Study (OCS) in Canada. Distribution of beliefs and willingness presented in the overall sample (*n =* 1677) and by past experience with anal cancer screening (not screened, *n =* 1006; screened, *n =* 671)

	Negative/Neutral (N)				Positive (P)	Strongly Positive (SP)
	Strongly Negative	Negative	Don’t Know	Neither		
***Normative Beliefs***						
**“My doctor thinks that I should get an exam for anal cancer”**						
Overall (*n =* 1677)	2%	24%	29%	14%	25%	6%
No cytology or anoscopy (not screened, *n =* 1006)	3%	29%	36%	15%	14%	2%
Has had cytology or anoscopy (screened, *n =* 671)	2%	18%	17%	12%	40%	12%
**“In general, people who are important to me would encourage me to get an exam for anal cancer**”						
Overall (*n =* 1677)	1%	9%	6%	9%	62%	13%
No cytology or anoscopy (not screened, *n =* 1006)	1%	10%	8%	11%	59%	11%
Has had cytology or anoscopy (screened, *n =* 671)	1%	7%	3%	7%	67%	15%
***Self-efficacy Beliefs***						
**“I can find out where to go to get an exam for anal cancer”**						
Overall (*n =* 1677)	< 1%	< 3%	4%	3%	71%	19%
No cytology or anoscopy (not screened, *n =* 1006)	< 1%	< 5%	5%	4%	70%	16%
Has had cytology or anoscopy (screened, *n =* 671)	1%	2%	1%	1%	73%	22%
**“I am confident that I could get an exam for anal cancer in the next year, if I chose to”**						
Overall (*n =* 1677)	< 1%	2%	5%	3%	69%	20%
No cytology or anoscopy (not screened, *n =* 1006)	< 1%	< 4%	7%	5%	68%	17%
Has had cytology or anoscopy (screened, *n =* 671)	< 1%	< 2%	2%	2%	71%	24%
***Behavioural Beliefs***						
**“I will feel pain during the procedure”**						
Overall (*n =* 1677)	2%	21%	11%	14%	45%	6%
No cytology or anoscopy (not screened, *n =* 1006)	1%	21%	16%	15%	43%	5%
Has had cytology or anoscopy (screened, *n =* 671)	4%	21%	4%	14%	49%	9%
**“I have a high chance of getting unpleasant short- term side effects, like pain or bleeding, after the procedure”**						
Overall (*n =* 1677)	3%	18%	19%	13%	43%	5%
No cytology or anoscopy (not screened, *n =* 1006)	2%	13%	25%	15%	42%	4%
Has had cytology or anoscopy (screened, *n =* 671)	5%	25%	9%	10%	45%	7%
**“If anal pre-cancer is found, I will be offered treatment”**						
Overall (*n =* 1677)	< 1%	< 1%	5%	< 2%	73%	20%
No cytology or anoscopy (not screened, *n =* 1006)	< 1%	< 2%	6%	3%	72%	19%
Has had cytology or anoscopy (screened, *n =* 671)	< 1%	< 2%	2%	1%	73%	23%
***Willingness to be screened***	**Very Unlikely**	**Unlikely**	**Undecided**		**Likely**	**Very Likely**
Overall (*n =* 1677)	2%	3%	5%		46%	44%
No cytology or anoscopy (not screened, *n =* 1006)	2%	4%	6%		49%	38%
Has had cytology or anoscopy (screened, *n =* 671)	2%	2%	2%		41%	53%

There were differences between men who have never been screened and those who have reported previous anal cancer screening by anal Pap or anoscopy (Table 2). Specifically, men who had never been screened were more likely to respond “don’t know” to the belief statements, particularly for the normative belief regarding a doctor’s recommendation (36% vs. 17% of those previously screened). Moreover, men who had never been screened were uncertain about likely outcomes, with 25% responding “don’t know” to “*I have a high chance of getting unpleasant short- term side effects, like pain or bleeding, after the procedure*” compared to 8% of those previously screened.

**Fig. 1:**
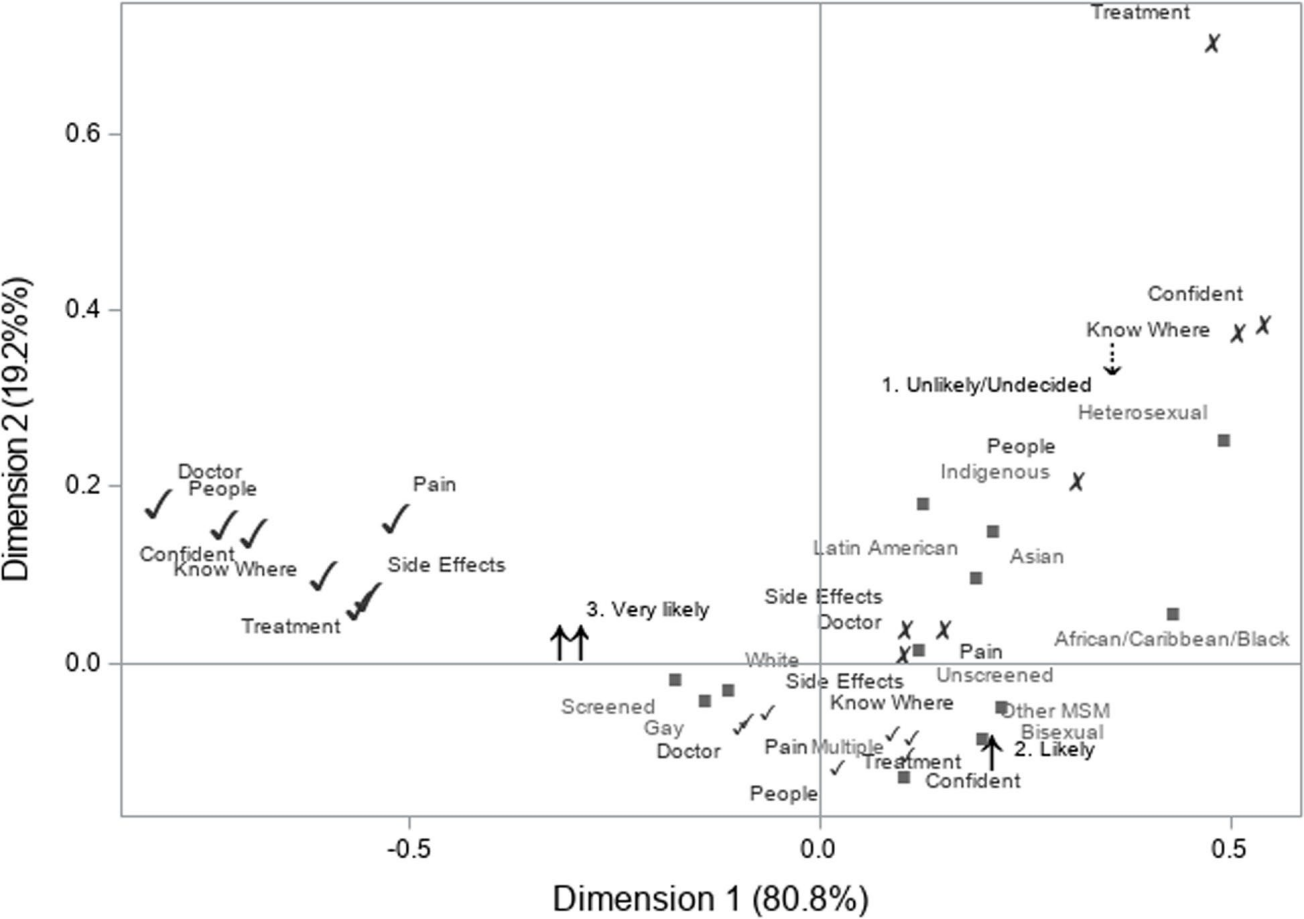
Correspondence analysis examining interrelationships between men’s beliefs regarding screening and willingness to undergo screening among men living with HIV attending HIV specialty care in 2016–2017 in the Ontario HIV Treatment Network Cohort Study (OCS) in Canada (large ✓: strongly positive beliefs regarding anal cancer screening; small✓: positive beliefs; ✗: negative/neutral beliefs; arrows: willingness to be screened; squares: supplementary variables—past screening, sexual orientation, race) Responses to belief statements labeled as: Doctor=“My *doctor* thinks that I should get an exam for anal cancer”; People=“In general, *people* who are important to me would encourage me to get an exam for anal cancer”; Know where=“I can find out *where* to go to get an exam for anal cancer”; Confident=“I am *confident* that I could get an exam for anal cancer in the next year, if I chose to”; Pai*n=*“I will feel *pain* during the procedure”; Side Effects=“I have a high chance of getting unpleasant short- term *side effects*, like pain or bleeding, after the procedure”; Treatment=“If anal pre-cancer is found, I will be offered *treatment*”. Willingness as response to “Think about what you might do in the next year. If anal cancer screening were offered to you, would you get an exam where a doctor or nurse inserts a swab (like a long, thin Q-tip) into your anus (“anal Pap test”)”: “*Very likely*”, “*Likely*” and combined “Undecided”, “Don’t know”, “Unlikely” and “Very unlikely” as “*Unlikely/Undecided*”. Supplementary variables: sexual orientation (*gay*, *bisexual*, other men who have sex with men (*MSM*), *heterosexual*); ethno-racial identity (*African/Caribbean/Black*, *Asian*, *Indigenous*, *Latin American*, *Multiple* selected, *white*); past screening (*Screened*, *Unscreened*)

In correspondence analysis, the first dimension (x-axis) represents the most influential relationships, whereby categories further from the origin represent the “largest deviation[s] from independence” [28]. Departures from the origin on the second dimension (y-axis) represent additional important associations. In our analysis (Fig. 1), distinct clusters were observed across the first dimension (x-axis), where strongly positive beliefs separated away from more moderate beliefs that clustered around the origin. This suggests that individuals are likely to have consistently strong positive beliefs across all the constructs and these beliefs were associated with men’s willingness to be screened for anal cancer. Specifically, strongly positive beliefs clustered with “very likely” to undergo future screening. Conversely, “indecision/unwillingness” was associated with negative and neutral beliefs. Sexual orientation, race, and past screening appear to be associated with men’s beliefs regarding screening; identifying as gay, white, and having been screened were most closely associated with being very willing to undergo screening and positive beliefs (Fig. 1).

The vast majority of men indicated they would be “very likely” (44%) or “likely” (46%) to undergo an “anal Pap test” if offered to them (Table 2). Multivariable proportional odds models were fit to examine factors associated with willingness to be screened (Table 3); there was no indication of the proportional odds assumption being violated. The global test for the hypothesis that past screening modifies the effect of men’s beliefs on willingness was not significant; all interaction terms were removed from the final multivariable model. In the multivariable model (Table 3), racialized men were less likely to undergo anal cancer screening if offered to them (aOR = 0.68; 95% CI: 0.53, 0.87) compared to white men. Specifically, African, Caribbean and Black men (aOR = 0.70; 95% CI: 0.48, 1.00) and Indigenous men (aOR = 0.56; 95% CI: 0.33, 0.97) indicated lower willingness than white men (Table 3). Sexual orientation was also associated with men’s willingness to be screened, where identifying as gay was positively associated with being more likely to undergo screening if offered (aOR = 1.68; 95% CI: 1.21, 2.32).

**Table 3:** Multivariable proportional odds models examining factors associated with willingness to be screened for anal cancer among men living with HIV attending HIV specialty care in 2016–2017 in the Ontario HIV Treatment Network Cohort Study (OCS) in Canada

	Unadjusted			Overall (***n =*** 1564)		
	% Undecided / Unwilling	% Likely	% Very Likely	aOR	95% CI	
**Orientation (ref: Heterosexual)**	25%	54%	21%			
Gay	7%	43%	50%	1.68	1.21	2.32
Bisexual	12%	56%	32%	1.30	0.81	2.08
Other men who have sex with men	13%	55%	32%	1.38	0.81	2.36
*** Contrast****: gay, bisexual and other men who have sex with men v. heterosexual men*				*1.44*	*1.02*	*2.02*
**Race (ref: White)**	8%	44%	48%			
African/Caribbean/Black	20%	57%	23%	0.70	0.48	1.00
Asian	16%	50%	34%	0.85	0.56	1.27
Indigenous	18%	43%	39%	0.56	0.33	0.97
Latin American	15%	51%	34%	0.64	0.40	1.03
Multiracial	8%	52%	40%	0.69	0.40	1.19
***Contrast:****Racialized men v. white men*				*0.68*	*0.53*	*0.87*
**Age (continuous per 10 years)**				1.08	0.98	1.20
**Previously screened by anal cytology or anoscopy**	8%	40%	52%	0.93	0.74	1.18
ref: No	13%	50%	37%			
**Familiar/knows a lot about HPV**	8%	40%	52%	1.05	0.83	1.32
Ref: Unfamiliar with HPV	14%	53%	33%			
**Knows someone with HPV-associated cancer**	6%	36%	58%	1.26	0.90	1.77
Ref: No	12%	48%	50%			
**Comfortable discussing anal health with family doctor**	9%	45%	46%	1.52	1.15	2.02
Ref: Not comfortable	20%	54%	27%			
**Self-reported diagnosis for anogenital warts**	7%	37%	56%	1.31	1.02	1.68
Ref: No	12%	50%	38%			
**Number of sexual recent partners (last 3 months; ref: None)**	13%	46%	41%			
One	10%	50%	40%	1.00	0.77	1.29
Two or more	7%	40%	53%	1.38	1.02	1.85
**Perceived risk for anal cancer (ref: No chance)**	18%	59%	23%			
Low chance	11%	44%	45%	1.61	1.21	2.14
Moderate	4%	43%	53%	2.11	1.45	3.08
High	4%	37%	59%	2.21	1.19	4.10
Certain I will get it or have it	6%	28%	66%	2.12	1.05	4.28
* Don’t know*	11%	45%	44%	2.32	1.58	3.86
**Beliefs regarding screening** **(ordinal from reference of negative/neutral beliefs to positive to strongly positive)**	*Proportion willing presented for strongly positive beliefs (ref: negative/neutral)*					
My doctor thinks that I should get an exam for anal cancer.	4% (12%)	13% (49%)	83% (39%)	1.29	1.04	1.60
In general, people who are important to me would encourage me to get an exam for anal cancer.	5% (21%)	14% (50%)	81% (29%)	1.79	1.47	2.18
I can find out where to go to get an exam for anal cancer.	5% (28%)	20% (51%)	74% (21%)	1.11	0.84	1.48
I am confident that I could get an exam for anal cancer in the next year, if I chose to.	5% (29%)	16% (51%)	79% (20%)	2.08	1.60	2.69
If anal pre-cancer is found, I will be offered treatment.	4% (36%)	24% (38%)	72% (26%)	1.48	1.12	1.96
I will feel pain during the procedure	9% (14%)	21% (50%)	70% (36%)	1.20	0.98	1.48
I have a high chance of getting unpleasant short- term side effects, like pain or bleeding, after the procedure.	6% (13%)	23% (49%)	71% (38%)	0.95	0.77	1.17

Positive normative beliefs were associated with men’s willingness to be screened (Table 3). Believing that “*people who are important to me would encourage me to get an exam for anal cancer*” was associated with higher willingness (aOR = 1.79; 95% CI: 1.47, 2.18), as was believing “*my doctor thinks that I should get an exam for anal cancer*” (aOR = 1.29; 95% CI: 1.04, 1.60). Positive beliefs regarding self-efficacy were also associated with willingness; specifically, agreeing with the statement “*I am confident that I could get an exam for anal cancer in the next year, if I chose to*” was associated with higher willingness (aOR = 2.08; 95% CI: 1.60, 2.69). Positive behavioral beliefs regarding treatment were associated with higher willingness (aOR = 1.48; 95% CI: 1.12, 1.96).

## Discussion

Among men attending HIV specialty clinics in Ontario, Canada, the vast majority (90%) indicated they would be likely or very likely to undergo anal cancer screening by “anal Pap test” in the next year if it were offered to them. Our findings suggest that high self-perceived risk for anal cancer and positive self-efficacy (i.e. feeling confident one could get screened) were most influential of men’s intention to be screened. Believing that treatment would be offered if anal pre-cancer were found was associated with higher willingness to be screened. Interestingly, the opinions of people important to the participants appeared to influence their willingness to be screened more strongly than their perception of a doctor’s recommendation, though both increased men’s willingness. Beliefs regarding possible side effects of screening do not seem to be deterrents to screening. There were notable differences in willingness to be screened by sexual orientation and ethno-racial identity after adjusting for perceived risk for anal cancer, beliefs regarding screening, age, familiarity with HPV, past experience with screening, and previous diagnosis with anogenital warts; heterosexual men and racialized men reported lower willingness to be screened.

Our study was conducted in a single-payer, publicly-funded healthcare setting, where medically-necessary services are free for patients at point of care [31]. Our results expand on previous studies examining intentions to undergo screening that were conducted in multi-payer healthcare settings [9, 11], where direct healthcare costs have been identified as significant barriers to participation [11]. In a study conducted in the United States, the proportion of men willing to be screened dropped significantly from 83% if freely available to 31% if it cost $150 US dollars [11]. In that study, men specifically listed cost, embarrassment, lack of information regarding anal cytology, and concerns about the accuracy of the test as reasons they were unwilling, whereas greater self-perceived risk for anal cancer was associated with willingness [11]. In the Multicenter AIDS Cohort Study (MACS) [9], 39% of the men living with HIV indicated they would possibly or likel*y* get screened in the next 6 months. The authors used the Andersen Model of Healthcare Utilization and identified that screening intentions were associated with predisposing (e.g. previously screened, number of sex partners), enabling (e.g. awareness of screening, health insurance), and need-related factors (e.g. HIV infection, history of anal warts) [9].

Accounting for similar factors in our study, as well as perceived risk and beliefs regarding screening, men from some racialized groups and heterosexual men reported being less willing to undergo anal cancer screening. In a recent study, similar disparities in actual receipt of anal cancer screening were observed, in that heterosexual men and men from some racialized groups were less likely to have discussed anal cancer screening or to have had digital anal rectal exams, anal Pap, or anoscopy [10]. Race is a social construct and proxy for factors that impact access to clinical care and health outcomes, including implicit biases and racism [32, 33]. Heterosexism in healthcare has also been identified as a significant barrier to anal cancer screening, whereby presumption of heterosexuality and anti-gay stigma may impede discussions of sexuality and concerns regarding anal health [34]. Moreover, the absence of clear guidelines for anal cancer screening has made counseling patients on anal cancer screening particularly challenging for healthcare providers [35, 36]. Altogether, these factors may lead to inequitable access to information that supports patients in their decision-making around anal cancer screening [35, 36].

Using the Theory of Planned Behavior, our study compliments previous studies by evaluating the role of beliefs regarding anal cancer screening on willingness to be screened, which can be used to inform patient-provider conversations and educational outreach. Ours was also one of the first to examine how past screening experience influences men’s beliefs and willingness to undergo screening in the future. Men who had not been screened were more likely to be uncertain about their doctor’s recommendation and about possible side effects of the procedures. It should be noted, however, that the influence of these beliefs on willingness to be screened was not modified by past screening per se. Past screening was also not independently associated with willingness, suggesting that beliefs regarding screening, rather than the past experience, itself, may be more predictive of willingness.

Strengths of the current study include being one of the largest to-date conducted in a single-payer, publicly-funded healthcare setting that broadly examines knowledge, experience and beliefs regarding HPV-associated diseases and their prevention. It is the first to use correspondence analysis to graphically examine the relationships between beliefs and willingness without imposing distributional assumptions. This method visually demonstrated the clustering and interrelatedness of beliefs regarding screening and willingness to be screened, as per the Theory of Planned Behavior. Moreover, this exploratory method was combined with proportional odds methodology to evaluate and appropriately model the ordinal nature of the Likert response format for men’s willingness to be screened.

However, there were limitations. Participants were from a convenience sample of men from a volunteer cohort of people receiving HIV care. The men in the OCS demonstrably engage in healthcare and receive care from HIV specialists who may be more familiar with the increased risk for anal cancer and provide screening for people living with HIV. Therefore, inference may not apply broadly to all men living with HIV and willingness to participate in screening may be lower in the general HIV-positive population. We had to combine distinct ethno-racial groups (e.g. Asian encompasses South Asian, East Asian and West Asian) due to small sample sizes for modeling considerations. The statements to assess men’s beliefs were framed around getting an “exam” for anal cancer, and therefore, we are unable to determine whether beliefs would differ according to screening modality. Moreover, the description of anoscopy does not include detailed information regarding the use of additional procedures to improve visualization (e.g., magnification, staining) or the potential for biopsy, which may impact willingness to undergo screening. Therefore, willingness to undergo high resolution anoscopy, specifically, may be overestimated from these data. Finally, we evaluated men’s willingness to undergo anal cancer screening if offered in the future which may not reflect actual acceptance of offered screening. Nonetheless, the Theory of Planned Behavior postulates that willingness and intention are immediate antecedents to participation in healthcare behaviors [27].

## Conclusions

Accessibility concerns should factor prominently when considering new screening approaches and clinical guidelines, as is anticipated for anal cancer screening. Factors such as cost and availability impact men’s willingness and participation in screening [11]; however, awareness and beliefs remain important and influential in both single-and multi-payer healthcare settings [9, 11]. Although men in our study generally had positive beliefs regarding anal cancer screening, our findings suggest that we may anticipate possible inequity given racialized men’s and heterosexual men’s reported lower willingness to be screened. To mitigate this, provider training and education for anal cancer screening for men living with HIV should address racism and stigma regarding anal health in healthcare; guidelines should be developed with structural interventions to address these issues [34]. Men living with HIV and their healthcare providers are also navigating complex health-related concerns and competing health priorities. Our finding that willingness may be influenced more by normative expectations regarding people important to men rather than a doctor’s recommendation suggests that developing materials to facilitate community-led conversations around anal cancer may be helpful to encourage screening participation. Community-derived and culturally-relevant materials, which normalize anal cancer screening [34], could also focus on men’s understanding of their risk and expectations of screening to facilitate participation across the diverse populations of men living with HIV.

## Supplementary Information

**Additional file 1.**

## Data Availability

The data that support the findings of this study are not publicly available to protect the privacy of the participants. However, all aggregated data from the OHTN Cohort Study (OCS) can be made available to researchers upon reasonable request and access to line-level data can be obtained through a request to the OCS Governance Committee. For more information, visit https://ohtncohortstudy.ca/research/. Requests to access data can be made by emailing the OCS coordinator at ocs@ohtn.on.ca.

## Abbreviations

aOR: Adjusted odds ratio
CI: Confidence interval
CIHR: Canadian Institutes of Health Research
CTN: Canadian HIV Trials Network
HIV: Human immunodeficiency virus
HPV: Human papillomavirus
HPV-SAVE: HPV-Screening, Ablation and Vaccine Evaluation
HRA: High resolution anoscopy
OCS: Ontario HIV Treatment Network Cohort Study
OHTN: Ontario HIV Treatment Network
Pap: Papanicolaou
