# Supplementary material for: Influence of previous experience with and beliefs regarding anal cancer screening on willingness to be screened among men living with HIV
Source: BMC Public Health. 2022 Dec 28;22:2444. doi: 10.1186/s12889-022-14471-4 (PMC9795733; doi:10.1186/s12889-022-14471-4)
Supplement: Supplementary file 1 — Additional file 1. [file 12889_2022_14471_MOESM1_ESM.docx]

**Supplemental Table 1: Summary of relevant questions and statements in the Ontario HIV Treatment Network Cohort Study questionnaire administered to men attending HIV specialty clinics in Ontario, Canada in 2016-2017**

| Questions and statements read to participants | Response categories |
| --- | --- |
| Have you heard of the human papillomavirus or “HPV”? | No  Heard the term but don’t know what it is  Heard the term and am familiar with it  Head the term and know a lot about it  Don’t know  Refuse to answer |
| *Interviewers read aloud a standardized summary about HPV and its importance for men living with HIV to provide all participants with basic knowledge of HPV prior to asking participants to perceive their risk for anal cancer.* | |
| HPV is sexually transmitted. Both women and men can get it from skin-to-skin contact during many types of sexual activities (e.g., oral, anal, vaginal sex). Most people will become infected with HPV at least once in their lifetime. Most of the time HPV has no symptoms so people don’t know they have it. HPV usually goes away on its own, but if it doesn’t, it can cause genital warts, anal warts, and several cancers, including cervical cancer, anal cancer, penile cancer, and some oral cancers. Men who are living with HIV are more likely to develop HPV-related cancers such as anal cancer than other men. People who have ever had receptive anal sex are also at higher risk of anal cancer, although one need not necessarily have had anal sex to get anal cancer. | |
| In your lifetime, what do you think your chance of getting anal cancer | No chance  Low chance  Moderate chance  High chance  Certain I will get it  Already had/have it |
| To the best of your knowledge, do you personally know someone who has had an HPV-related cancer (other than yourself)? Remember, some cancers caused by HPV include cervical cancer, anal cancer, penile cancer, and some oral cancers. | Yes  No  Don’t know  Refused |
| How comfortable are you discussing health issues relating to your anus with your family doctor? | Very comfortable  Comfortable  Neither comfortable nor uncomfortable  Uncomfortable  Very uncomfortable  Don’t know  Refused |
| Have you ever had the following types of anal cancer screening?   1. an exam where a doctor or nurse inserted a swab (like a long, thin Q-tip) into your anus (“anal Pap test”). 2. an “anoscopy ” where a doctor inserts a device called an anoscope into your anus. The anoscope goes in about 5 cm (2 inches) and it is about 2.5 cm (1 inch) wide. Using an anoscope, the doctor gets a detailed look at the tissue inside | Yes  No  Don’t know  Refused |
| Think about what you might do in the next year. If anal cancer screening were offered to you, would you get …   1. an exam where a doctor or nurse inserts a swab (like a long, thin Q-tip) into your anus (“anal Pap test”). 2. an “anoscopy ” where a doctor inserts a device called an anoscope into your anus. The anoscope goes in about 5cm (2 inches) and it is about 2.5 (1 inch) wide. Using an anoscope, the doctor can get a detailed look at the tissue inside. | Very likely  Likely  Undecided  Unlikely  Very unlikely  Don’t know  Refused |
| Please tell me to what extent you agree or disagree with the following statements about getting a test or exam for anal cancer.   1. My doctor thinks that I should get an exam for anal cancer. 2. In general, people who are important to me would encourage me to get an exam for anal cancer. 3. I will feel pain during the procedure. 4. If anal pre-cancer is found, I will be offered treatment. 5. I have a high chance of getting unpleasant short- term side effects, like pain or bleeding, after the procedure. 6. I can find out where to go to get an exam for anal cancer. 7. I am confident that I could get an exam for anal cancer in the next year, if I chose to. | Strongly disagree  Disagree  Neither agree nor disagree  Agree  Strongly agree  Don’t know  Refused |
